# Quorum Sensing in Fungi: Q&A

**DOI:** 10.1371/journal.ppat.1002301

**Published:** 2011-10-27

**Authors:** Hiten D. Madhani

**Affiliations:** Department of Biochemistry and Biophysics, University of California San Francisco, San Francisco, California, United States of America; Duke University Medical Center, United States of America

## I know that the accumulation of certain molecules called “quorum-sensing factors” in bacteria, as cultures grow dense, can control group behavior and that these include virulence traits and the formation of drug-resistant microbial biofilms. I am familiar with some of the molecules that mediate the sensing of organism density such as homoserine lactones and peptides. However, I don't know about such systems in fungi. What types of molecules are involved?

**Figure 1:**
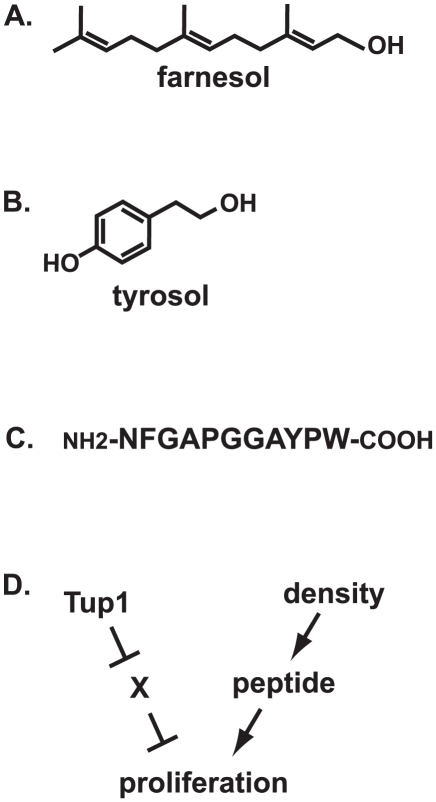
Fungal quorum-sensing molecules and mechanisms. (A) Structure of farnesol; (B) structure of tyrosol; (C) sequence of mature Qsp1 peptide; (D) model for Qsp1 action in *C. neoformans var. neoformans*.

In the human commensal and pathogenic fungus *Candida albicans*, two molecules have been described: farnesol and tyrosol [1], [2]. Farnesol is a sesquiterpine alcohol, which is an alcohol made up of three isoprene units (Figure 1A). Tyrosol is an alcohol related to the amino acid tyrosine (Figure 1B). A completely different type of molecule, a peptide (Figure 1C), is made by the human opportunistic yeast *Cryptococcus neoformans*.

## How are farnesol and tyrosol made by *C. albicans*?

Farnesol pyrophosphate is an intermediate in sterol biosynthesis, and there is evidence that this is the source produced by *C. albicans*. However, the subsequent enzymatic pathway involved and the export pathways (if any) are unknown. Tyrosol synthesis requires an intact aromatic amino acid biosynthetic pathway, but again the detailed enzymology remains to be worked out [1].

## Is the peptide in *C. neoformans* synthesized by nonribosomal synthases or by the ribosome?

Unlike some bacterial quorum-sensing peptides that are synthesized by nonribosomal synthases, the Cryptococcal peptide is synthesized from a larger precursor and is apparently processed and exported [3]. The enzymes and mechanisms are unknown.

## Do these molecules function as sensors of culture density?

One definition of quorum sensing requires that the signal be synthesized at a fixed rate; thus, its concentration would be proportional to the concentration of the microbe in a liquid culture. Tyrosol is regulated by environmental conditions [4], and the *QSP1* gene that encodes the Cryptococcal peptide is directly regulated by a signal-regulated DNA-binding regulator [5]. Therefore, these factors may be more accurately thought of as auto-regulatory molecules rather than pure sensors of culture density.

## Does the accumulation of these factors promote cell division?

Tyrosol can overcome the lag experienced by sparse cultures of *C. albicans* [1]. Microarray expression analysis suggests that tyrosol induces the expression of genes involved in DNA replication [1]. The Cryptococcal peptide can also overcome an inhibition of growth at low culture density, but the story is more complex: only mutants lacking the Tup1 corepressor display a dependency on the peptide for growth [3]. One model to explain this observation is that Tup1 (presumably acting through a sequence-specific DNA binding repressor) turns off an inhibitor of growth that is redundantly inhibited by the quorum-sensing system (Figure 1D). Thus, the quorum system may only be required for growth normally when repression of a target by Tup1 is relieved in response to environmental signals.

## I know that some bacteria regulate biofilm formation in response to quorum-sensing signals. Does this happen in fungi?

Yes. *C. albicans* biofilm formation is inhibited by farnesol [6]. This has been suggested to regulate the extent of biofilm formation. Microarray analysis indicates that farnesol activates hyphal-specific gene expression while inhibiting the expression of a cell surface hydrophobin [7]. Whether endogenous farnesol production controls biofilm formation will require mutants specifically defective in its synthesis.

## Many pathogenic fungi are dimorphic, switching between yeast and hyphal forms. Does quorum-sensing control this transition?

Indeed, this is how farnesol was isolated: dense cultures of *C. albicans* display a reduced propensity for the yeast-to-hyphal switch, and this is mediated by the accumulation of farnesol. This may limit nutrient foraging behavior under conditions that are nutrient replete.

## Do we know anything about the receptors or downstream signaling pathways that respond to fungal quorum-sensing molecules?

We are just beginning to answer this question. A receptor histidine kinase homolog called Chk1 in *C. albicans* has been implicated in the inhibition of hyphal growth by farnesol, but the story is not simple [8]. More recently, it has been shown that that farnesol reception requires a signaling pathway that includes the conserved small GTPase Ras and its downstream effector, adenylate cyclase, and a DNA-binding repressor called Nrg1 [9]–[11]. We know nothing of the reception mechanism for tyrosol and the Cryptococcal peptide.

## You have told me about *C. albicans* and *C. neoformans*, but is there evidence for quorum-sensing phenomenon in other pathogenic fungi?

Yes [12]. Inoculum size affects the yeast–hyphal transition in the human pathogens *Histoplasma capsulatum* and *Mucor rouxii*. The factors involved remain to be identified.

## Is there evidence for cross-species communication via fungal quorum-sensing molecules?

It has been reported that cocultivation of *C. albicans* and the nonpathogenic filamentous fungus *Aspergillus nidulans* results in farnesol-dependent inhibition of growth of the latter [13]. Moreover, farnesol can inhibit growth, biofilm formation, and virulence factors by some bacteria [14]–[17], which could be relevant for the lifestyle of *C. albicans* as a human gut commensal.

## Do quorum-sensing signaling mechanisms play a role in pathogenesis?

Given the abundant evidence that the yeast-to-hyphal switch is important for *C. albicans* virulence, farnesol production could play a role in pathogenesis, but this remains to be proven.

## Where do we go from here?

Identifying additional quorum-sensing molecules in more pathogenic fungi would be a good start. In *C. albicians* and *C. neoformans*, defining the molecules responsible for export as well as receptors and intracellular signal transduction mechanisms would allow the construction of mutants to test the biological role of these systems.

## References

[1] Chen H, Fujita M, Feng Q, Clardy J, Fink GR (2004). Tyrosol is a quorum-sensing molecule in *Candida albicans*.. Proc Natl Acad Sci U S A.

[2] Hornby JM, Jensen EC, Lisec AD, Tasto JJ, Jahnke B (2001). Quorum sensing in the dimorphic fungus *Candida albicans* is mediated by farnesol.. Appl Environ Microbiol.

[3] Lee H, Chang YC, Nardone G, Kwon-Chung KJ (2007). TUP1 disruption in *Cryptococcus neoformans* uncovers a peptide-mediated density-dependent growth phenomenon that mimics quorum sensing.. Mol Microbiol.

[4] Ghosh S, Kebaara BW, Atkin AL, Nickerson KW (2008). Regulation of aromatic alcohol production in *Candida albicans*.. Appl Environ Microbiol.

[5] Chun CD, Brown JC, Madhani HD (2011). A major role for capsule-independent phagocytosis-inhibitory mechanisms in mammalian infection by *Cryptococcus neoformans*.. Cell Host Microbe.

[6] Ramage G, Saville SP, Wickes BL, Lopez-Ribot JL (2002). Inhibition of *Candida albicans* biofilm formation by farnesol, a quorum-sensing molecule.. Appl Environ Microbiol.

[7] Cao YY, Cao YB, Xu Z, Ying K, Li Y (2005). cDNA microarray analysis of differential gene expression in *Candida albicans* biofilm exposed to farnesol.. Antimicrob Agents Chemother.

[8] Kruppa M, Krom BP, Chauhan N, Bambach AV, Cihlar RL (2004). The two-component signal transduction protein Chk1p regulates quorum sensing in *Candida albicans*.. Eukaryot Cell.

[9] Deveau A, Piispanen AE, Jackson AA, Hogan DA (2010). Farnesol induces hydrogen peroxide resistance in *Candida albicans* yeast by inhibiting the Ras-cyclic AMP signaling pathway.. Eukaryot Cell.

[10] Hall RA, Turner KJ, Chaloupka J, Cottier F, De Sordi L (2011). The quorum-sensing molecules farnesol/homoserine lactone and dodecanol operate via distinct modes of action in *Candida albicans*.. Eukaryot Cell.

[11] Lu Y, Su C, Wang A, Liu H (2011). Hyphal development in *Candida albicans* requires two temporally linked changes in promoter chromatin for initiation and maintenance.. PLoS Biol.

[12] Hogan DA (2006). Talking to themselves: autoregulation and quorum sensing in fungi.. Eukaryot Cell.

[13] Semighini CP, Hornby JM, Dumitru R, Nickerson KW, Harris SD (2006). Farnesol-induced apoptosis in *Aspergillus nidulans* reveals a possible mechanism for antagonistic interactions between fungi.. Mol Microbiol.

[14] Jabra-Rizk MA, Meiller TF, James CE, Shirtliff ME (2006). Effect of farnesol on *Staphylococcus aureus* biofilm formation and antimicrobial susceptibility.. Antimicrob Agents Chemother.

[15] Koo H, Hayacibara MF, Schobel BD, Cury JA, Rosalen PL (2003). Inhibition of *Streptococcus mutans* biofilm accumulation and polysaccharide production by apigenin and *tt*-farnesol.. J Antimicrob Chemother.

[16] Cugini C, Calfee MW, Farrow JM, Morales DK, Pesci EC (2007). Farnesol, a common sesquiterpene, inhibits PQS production in *Pseudomonas aeruginosa*.. Mol Microbiol.

[17] Cugini C, Morales DK, Hogan DA (2010). Candida albicans-produced farnesol stimulates *Pseudomonas* quinolone signal production in LasR-defective *Pseudomonas aeruginosa* strains.. Microbiology.

